# Dietary Supplementation With High Fiber Alleviates Oxidative Stress and Inflammatory Responses Caused by Severe Sepsis in Mice Without Altering Microbiome Diversity

**DOI:** 10.3389/fphys.2018.01929

**Published:** 2019-01-18

**Authors:** Yuanyuan Zhang, Aili Dong, Keliang Xie, Yonghao Yu

**Affiliations:** Department of Anesthesiology, Tianjin Medical University General Hospital, Tianjin, China

**Keywords:** fiber, sepsis, mitochondrion, HO-1, Nrf2

## Abstract

In this study, we demonstrated the effects of a high-fiber diet on intestinal lesions, oxidative stress and systemic inflammation in a murine model of endotoxemia. C57BL/6 mice were randomly assigned to four groups: the control group (CONTROL), which received a commercial normal-fiber rodent diet comprising normal fiber; a CLP group, which received a commercial normal-fiber rodent diet and underwent caecal ligation puncture (CLP); a high-fiber group (HFG), which received a commercial high-fiber rodent diet; and a high fiber + CLP group (HFCLP) which received a commercial high-fiber rodent diet and underwent CLP (30%). The sepsis model was created via CLP after 2 weeks of dietary intervention. Notably, dietary high-fiber supplementation in HFCLP group improved survival rates and reduced bacterial loads, compared with CLP alone. In the HFCLP group, dietary fiber supplementation decreased the serum concentrations of pro-inflammatory cytokines such as tumor necrosis factor-α (TNF-α), interleukin 6 (IL-6) and high-mobility group protein 1 (HMG-1) but raised the concentration of interleukin 10 (IL-10), compared with the levels in CLP mice. Meanwhile, high-fiber supplementation increased the relative proportions of *Akkermansia* and *Lachnospiraceae*. These data show that dietary high-fiber supplementation may be therapeutic for sepsis-induced lesions.

## Introduction

In 2011, more than $20 billion in hospital costs in the United States were attributed to sepsis (Singer et al., 2016), a life-threatening organ pathology caused by a dysregulated host response to infection. Sepsis pathogenesis is typically classified as an initial pro-inflammatory phase, followed by an anti-inflammatory or immunosuppressive phase (Haak et al., 2018; Shankar Hari and Summers, 2018). During the last 30 years, researchers have investigated a number of unsuccessful immunotherapeutic strategies aimed at circumventing the unregulated pro-inflammatory host response during the initial phases of sepsis. However, most of these strategies focused on the cascade of pro-inflammatory cytokines, including tumor necrosis factor α (TNF-α), interleukin (IL)-1 and high-mobility group box 1 (HMGB1), which have all been shown to be of little practical therapeutic value.

Dietary modifications can affect systemic inflammation via changes in the gut microbiota (Kau et al., 2011; Tilg and Moschen, 2015). Generally, fiber is classified as either ‘fermentable’ or ‘non-fermentable’ (i.e., resistant), and studies have investigated the anti-inflammatory properties and mechanisms of the former type (Wedlake et al., 2014; Simpson and Campbell, 2015). By contrast, the protective anti-inflammatory properties of cellulose, a non-fermentable fiber, have yet to be elucidated. Dietary fiber has well-documented anti-inflammatory characteristics, which can be partly attributed to fiber-induced actions on the gut microbiota (Kuo, 2013; Simpson and Campbell, 2015). Morowitz et al. (2017) demonstrated that the benefits associated with dietary cellulose intake correlate with enrichment of the gut microbiome taxon *Akkermansia*, a genus typically associated with improved metabolic health. This finding led us to hypothesize that supplementation with cellulose would enhance survival in murine sepsis models by reducing intestinal lesions, modulating oxidative stress and reducing systemic inflammation.

In this investigation, we demonstrated the effects of a high-fiber diet on intestinal lesions, oxidative stress and systemic inflammation in a murine model of endotoxemia.

## Materials and Methods

### Use and Care of Animals

All animal investigations were approved by the Tianjin Medical University General Hospital, Tianjin, China. Animals were cared for in accordance with the Chinese guidelines for animal use and treatment. C57BL/6 mice were randomly assigned to four groups (*n* = 20 each): control (CONTROL), which was fed a commercial normal-fiber rodent diet (5% cellulose); CLP, which was also fed a commercial normal-fiber rodent diet and underwent caecal ligation and puncture (CLP); high-fiber (HFG), which was fed a commercial high-fiber rodent diet (30% cellulose); and high-fiber + CLP (HFCLP), which was fed a commercial high-fiber rodent diet and subjected to CLP. The mouse weights were monitored daily. After a 2-week dietary intervention, a sepsis model was created by CLP according to our previous report (Yu et al., 2017). Mice in all groups were subjected to hypodermic peritoneal injection with 1 mL of a 0.9% saline solution immediately after the operation. The resulting lavage fluid was serially diluted with sterile saline, and 100-μL aliquots of the dilutions were placed on agar plates and incubated at 37°C for 16 h. Subsequently, colony-forming units (CFUs) in the samples of peritoneal lavage fluid were calculated in accordance with previous studies (Morowitz et al., 2017). Serum, tissue and fecal samples were stored at −80°C for further analysis.

### Morphology Analysis of Intestinal Tissue

The small intestines of all mice were fixed in 10% paraformaldehyde, embedded in paraffin and stained with haematoxylin and eosin (HE). The disease scores were then rated by two pathologists who were blind to the experimental design and grouping to assess the extent of intestinal lesions (Shrum et al., 2014; Yu et al., 2017).

### Measurement of Oxidative Products, Antioxidant Enzymes and Inflammatory Cytokines

Twenty-four hours postoperatively, 10-mL blood samples were collected from 8 mice per group and centrifuged at 3500 rpm for 8 min. Subsequently, serum samples were collected and stored at −80°C, after which the levels of oxidative products (e.g., malondialdehyde [MDA] and 8-iso-15(S)-prostaglandin F2α [8-iso-PGF2α]) were detected using a commercial kit (Nanjing Jiancheng Bio Co., Ltd., Nanjing, China). Catalase (CAT) and superoxide dismutase (SOD) activities in the sera were also detected using kits (Nanjing Jiancheng Co., Ltd., Nanjing, China) according to the manufacturer’s instructions. Enzyme-linked immunosorbent assay (ELISA) kits were used to determine the serum concentrations of TNF-α, IL-6, IL-10 (R&D Systems) and HMGB1 (Nanjing Jiancheng Co., Ltd., Nanjing, China) in accordance with the manufacturers’ instructions (Yu et al., 2017).

### Real-Time Quantitative PCR

The levels of nuclear factor (erythroid-derived 2)-like 2 (Nrf2) and heme oxygenase-1 (HO-1) mRNA were detected using real-time quantitative PCR. Expression of *Gapdh* mRNA was used as a reference. The following gene-specific primer sequences were used: Nrf2-F 5′-CGACAGAAACCTCCATCTACTGAA-3′, Nrf2-R 5′-CCTCATCACGTAACATGCTGAAG-3′; HO-1-F 5′-ACAGATGGCGTCACTTCG-3′, HO-1-R 5′-TGAGGACCCACTGGAGGA-3′; GAPDH-F 5′-CATCACTGCCACCCAGAAGAC-3′, GAPDH-R 5′-CCAGTGAGCTTCCCGTTCAG-3′ (Yu et al., 2017).

### Sequencing and Analysis of Bacterial 16S rRNA Genes

Total DNA was extracted from fecal samples and purified, and the V4 regions of 16S rRNA genes were amplified using specific primers (515F-806R). All PCR reactions were performed using Phusion^®^High-Fidelity PCR Master Mix (New England Biolabs, Ipswich, MA, United States). Sequencing was performed on an Illumina MiSeq device (Illumina, Inc., San Diego, CA, United States), and QIIME (v1.9) was used to demultiplex the raw sequence reads. UPARSE (v8.0) was then used to filter the reads for quality. Sequences with a similarity >97% were classified in the same operational taxonomic unit (OTU). UCLUST and Greengenes reference database (v13.8) were then used to assign taxonomies to the predicted OTUs. Alpha diversity and QIIME (Version 1.7.0) were used to analyze the complexity of each sample.

### Statistical Analysis

Mouse survival rates are expressed as percentages (%). Other data are presented as means ± standard deviations (SDs). The log-rank (Mantel–Cox) test was used to evaluate differences in survival rates between the groups; the unpaired t-test or Mann–Whitney test was also used if the results were approximately normally distributed (e.g., Gaussian distribution) or not normally distributed, respectively. A *P*-value <0.05 was considered to indicate a statistically significant difference. The statistical analyses were performed using SPSS, version 21.0 (IBM Corp., Armonk, NY, United States).

**FIGURE 1:**
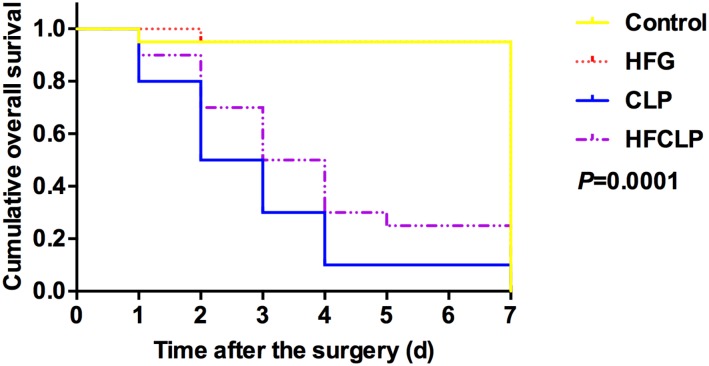
Effect of dietary fiber supplementation on the survival rates of mice. The HFG group overlapped with the CONTROL group after day 2.

**FIGURE 2:**
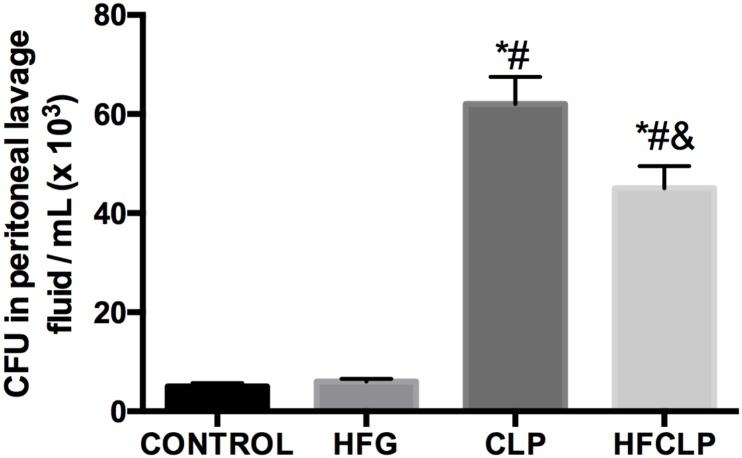
Effect of dietary fiber supplementation on CFU counts in the peritoneal lavage fluid (*n* = 8). ^∗^*P* < 0.05 vs. the CONTROL group, ^#^*P* < 0.05 vs. the HFG group and ^&^*P* < 0.05 vs. the CLP group.

## Results

### Survival Rate and Bacterial Load

Figure 1 suggests that a minimal number of mice died in each of these groups, the survival rates in the CLP and HFCLP mouse groups decreased significantly (*P* < 0.05). However, the mouse groups subject to dietary high-fiber supplementation exhibited an enhanced sepsis survival rate (*P* < 0.05). We further analyzed the CFUs in peritoneal lavage fluid and found significantly higher numbers in the CLP and HFCLP groups (*P* < 0.05). Notably, the HFCLP group exhibited a marked reduction in CFUs, compared to the CLP group (*P* < 0.05) (Figure 2).

**FIGURE 3:**
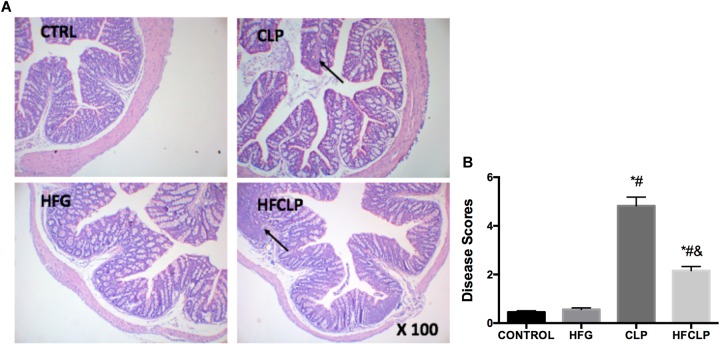
Effects of dietary fiber supplementation on pathological intestinal changes. **(A)** HE staining of intestinal tissues (x 100) and **(B)** histopathological disease scores of the intestines. ^∗^*P* < 0.05 vs. the CONTROL group, ^#^*P* < 0.05 vs. the HFG group, and ^&^*P* < 0.05 vs. the CLP group.

### Small Intestinal Morphology and Disease Scores

To determine the severity of intestinal lesions, the small intestines were subjected to HE staining, and appropriate histopathological scores were assigned to rate the severities of the observed intestinal injuries (Figure 3). In the CONTROL and HFG groups, the intestinal mucosa did not exhibit any abnormal morphological changes. However, shortening and atrophy of the intestinal mucosal villi were observed in the CLP and HFCLP groups. The Intestinal lesions were less severe in the HFCLP group than in the CLP group (*P* < 0.05). In addition, the intestinal disease scores of mice in the CLP and HFCLP groups were much higher than those in the CONTROL and HFG groups (*P* < 0.05).

**FIGURE 4:**
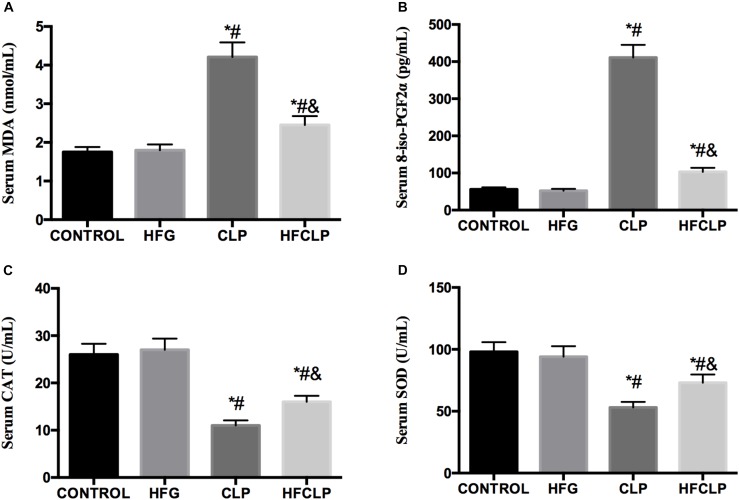
Effects of dietary fiber supplementation on the serum levels of **(A)** MDA, **(B)** 8-iso-PGF2α, **(C)** CAT and **(D)** SOD. ^∗^*P* < 0.05 vs. the CONTROL group, ^#^*P* < 0.05 vs. the HFG group, and ^&^*P* < 0.05 vs. the CLP group.

### Oxidative Products and Antioxidative Enzymes

Figure 4 indicates that the levels of MDA and 8-iso-PGF2α were higher in the CLP and HFCLP groups than in the CONTROL and HFG groups (*P* < 0.05). Meanwhile, the levels of both oxidative products were lower in the HFCLP group than in the CLP group (*P* < 0.05). However, the activities of the anti-oxidative enzymes CAT and SOD were lower in the CLP and HFCLP groups than in the CONTROL and HFG groups (*P* < 0.05). Moreover, the activities of both enzymes were higher in the HFCLP group than in the CLP group (*P* < 0.05).

**FIGURE 5:**
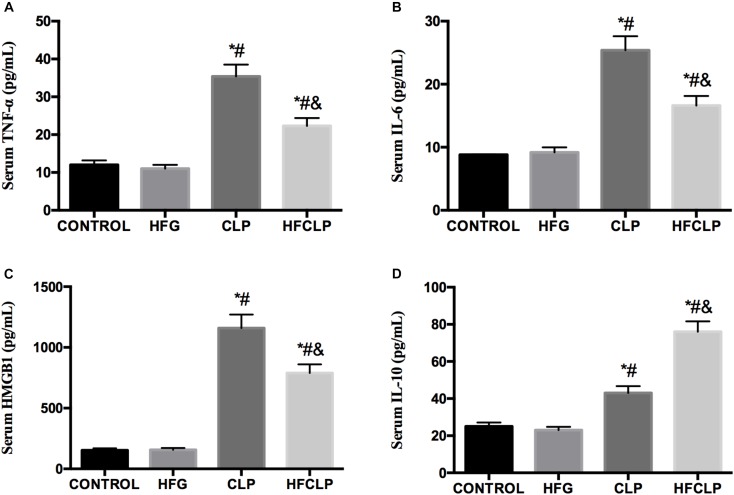
Effect of dietary fiber supplementation on the serum levels of the cytokines **(A)** TNF-α, **(B)** IL-6, **(C)** HMGB1, and **(D)** IL-10. ^∗^*P* < 0.05 vs. the CONTROL group, ^#^*P* < 0.05 vs. the HFG group, and ^&^*P* < 0.05 vs. the CLP group.

**FIGURE 6:**
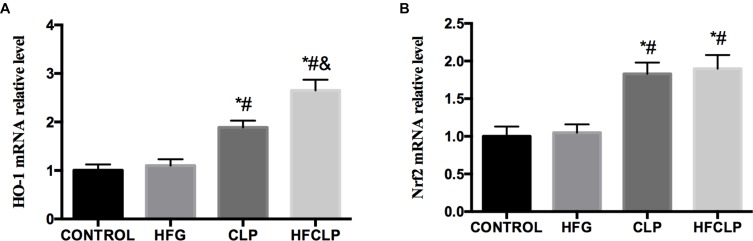
Effects of dietary fibre supplementation on the levels of **(A)** HO-1 and **(B)** Nrf2 mRNA. ^∗^*P* < 0.05 vs. the CONTROL group, ^#^*P* < 0.05 vs. the HFG group and ^&^*P* < 0.05 vs. the CLP group.

### Serum Inflammatory Cytokines

Next, the serum levels of inflammatory cytokines were investigated. Figure 5 demonstrates that the serum concentrations of TNF-α, IL-6 and HMGB1 were significantly increased in the CLP group, compared to the CONTROL and HFG groups (*P* < 0.05). However, the dietary fiber supplementation provided to the HFCLP group reduced TNF-α, IL-6 and HMGB1 levels markedly, compared to those in the CLP group (*P* < 0.05). The CLP and HFCLP groups exhibited significantly higher serum IL-10 concentrations relative to those in the CONTROL and HFG groups (*P* < 0.05). Furthermore, the serum IL-10 level was significantly lower in the CLP group than in the HFCLP group (*P* < 0.05).

### HO-1 and HMGB1 Expression

The levels of HO-1 mRNA were significantly higher in the CLP and HFCLP groups than in the CONTROL and HFG groups (*P* < 0.05). Furthermore, the level of HO-1 mRNA was higher in the HFCLP group, compared to the CLP group (*P* < 0.05) (Figure 6). Similarly, the levels of Nrf2 mRNA were significantly higher in the CLP and HFCLP groups than in the CONTROL and HFG groups (*P* < 0.05). However, no significant difference in this transcript was observed between the CLP and HFCLP groups.

### Microbial Diversity in the Fecal Samples

**FIGURE 7:**
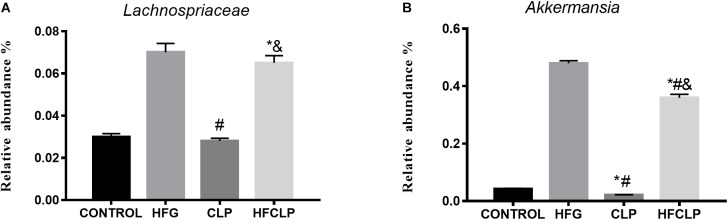
Relative abundances of the microbial taxa predicted to be enriched or depleted after dietary fibre supplementation. The indicated taxa **(A)** *Lachnospiraceae* and **(B)** *Akkermansia* had relatively high abundances across all sample groups. ^∗^*P* < 0.05 vs. the CONTROL group, ^#^*P* < 0.05 vs. the HFG group and ^&^*P* < 0.05 vs. the CLP group.

Bacterial 16S rRNA gene sequencing was used to profile the gut microbiota in mice from each group. Notably, no statistical differences in alpha diversity were observed among the four groups (Table 1). However, significant between-group differences were observed in the community compositions of the fecal samples. In particular, samples collected from the HFG and HFCLP groups contained highly abundant bacteria from the family Lachnospiraceae, which are typically associated with a healthy colon (*P* < 0.05). Meanwhile, the relative abundance of *Akkermansia*, a bacterial genus with known health-enhancing characteristics, was significantly higher in the HFG and HFCLP groups (*P* < 0.05) (Figure 7).

## Discussion

Septic shock is a frequent cause of mortality in critical patients (Mayr et al., 2014; Rickard et al., 2014). Previous reports have shown that sepsis morbidity might result from an extreme pro-inflammatory response and/or extreme anti-inflammatory response which generates a state of immunosuppression (Mayr et al., 2014; Rickard et al., 2014). In this investigation, we successfully generated a sepsis model and demonstrated that dietary high-fiber supplementation led to an improved survival rate with lower bacterial loading, compared to CLP treatment alone. In addition, supplementation with a high-fiber dietalleviated intestinal lesions and oxidative injuries, thereby enhancing survival and reducing the serum levels of pro-inflammatory cytokines in a CLP-induced murine sepsis model.

**Table 1:** Microbial alpha diversity in the collected fecal samples.

Items	CONTROL	HFG	CLP	HFCLP
OTU	1276	1143	1212	1098
Chao1	1035.65	1022.74	1084.83	1044.67
Ace	1120.13	1069.18	1132.46	1106.94
Shannon	5.21	4.92	5.09	5.15
Simpson	0.88	0.91	0.89	0.89

The generation of pro- and anti-inflammatory mechanisms has been suggested to represent a vital stage in sepsis survival (Munford and Pugin, 2001; Rickard et al., 2014). In our study, mice with severe CLP-induced sepsis in the CLP and HFCLP groups exhibited more severe intestinal injuries, compared to untreated mice. Excessive cytokines secretion and elevated oxidative species levels underpin the pathogenesis of sepsis (Xie et al., 2014). To observe the effects of dietary fiber supplementation on intestinal lesions induced by severe sepsis, the concentrations of inflammatory factors (e.g., pro- and anti-inflammatory cytokines) were monitored. Similarly, dietary fiber supplementation was shown to reduce the concentrations of pro-inflammatory cytokines, including TNF-α, IL-6 and HMGB1, and increase the concentration of IL-10 in sera from HFCLP mice relative to sera from the CLP group. Previous investigations have demonstrated that HMGB1 is a useful marker of severe sepsis, and several reports have demonstrated that once activated and secreted into the extracellular milieu, this cytokine can mediate sepsis-related inflammatory responses (Wang et al., 2014; Stevens et al., 2017). Consistent with earlier studies (Bae, 2012; Nogueira-Machado and de Oliveira Volpe, 2012; Cho and Choi, 2014), our investigation demonstrated a correlation of the HMGB1 level with intestinal lesion severity. These data demonstrate that dietary fiber supplementation improves the clinical outcomes of mice subjected to sepsis.

The transcription factor Nrf2 is a key regulator of suitable antioxidant and anti-inflammatory responses (Vriend and Reiter, 2015; Ren et al., 2018). This investigation demonstrated greater mRNA levels of HO-1 and Nrf2 in mice subjected to CLP injection relative to normal mice. However, even higher levels were observed in septic mice subjected to the dietary high-fiber intervention. Severe sepsis can cause disintegration of the intestinal tight junctions, resulting in systemic inflammation and oxidative stress (Ren et al., 2018). At this time, Nrf2 may be activated to translocate from the cytoplasm to the nucleus, where it binds to the ARE gene and thereby regulates the expression of SOD and CAT (Liu et al., 2014). HO-1, which is generated downstream of Nrf2, exerts beneficial actions against and thus downregulates pro-inflammatory responses (Yu et al., 2009; Vijayan et al., 2011; Bortscher et al., 2012).

The configuration of the gut microbiota has been shown to influence therapeutic responses in a variety of clinical conditions, including cancer and diabetes (Taur et al., 2014; Forslund et al., 2015; Vetizou et al., 2015). To date, however, clinical investigations of sepsis have not considered the status of the gut microbiota (i.e., they did not assess individual gut microbiota species present within the gut over the disease period). Diet is known to represent a robust connection between the gut microbiota and immune function (Kau et al., 2011; Tilg and Moschen, 2015). It would seem that this association is relevant to sepsis survival. In this investigation, the high-fiber intervention partly protected against systemic inflammation and mortality in a murine sepsis model. Earlier work by Peck et al. indicated that calorie restriction also enhanced survival in mice challenged with *S. typhimurium* (Peck et al., 1992). In this study, mice also exhibited clear alterations in gut microbiota, particularly an enrichment of the genus *Akkermansia*. These anaerobic micro-organisms are typically found in both human and rodent gut microbiomes, and their abundance in humans has been shown to inversely correlate with body weight and inflammatory activity in patients with inflammatory bowel disease (Png et al., 2010; Santacruz et al., 2010).

## Conclusion

This study has demonstrated that dietary supplementation with high fiber alleviates intestinal injuries. The mechanism of action is thought to be partially attributable to modifications of both the microbiota and host physiology by fiber supplementation, which thereby permit an appropriate and survival-promoting inflammatory response to the injury. Possibly, an improved comprehension of the correlations between the diet, microbiota and systemic pathology could lead to novel diet-based therapeutic approaches for sepsis. However, additional investigations are needed to assess the potential benefits of an intervention comprising dietary high-fiber supplementation for the treatment of severe sepsis.

## Data Availability

All the data are available at YZ (yzhang10@tmu.edu) upon request.

## Author Contributions

YZ and YY designed the research and proofread the manuscript. AD and KX carried out the study. AD, KX, and YY analyzed the data. YZ wrote the manuscript.

## Conflict of Interest Statement

The authors declare that the research was conducted in the absence of any commercial or financial relationships that could be construed as a potential conflict of interest. The reviewer HN and handling Editor declared their shared affiliation.
